# Pulse and legume consumption is associated with a more optimal nutrient intake and a higher EAT-Lancet index in a representative UK population

**DOI:** 10.1007/s00394-025-03611-2

**Published:** 2025-03-26

**Authors:** Yankho Kaimila, Oyinkansola A. Olotu, Miriam E. Clegg, Kim G. Jackson, Julie A. Lovegrove

**Affiliations:** 1 Hugh Sinclair Unit of Human Nutrition, University of Reading, Harry Nursten Building, Pepper Lane, Reading, RG6 6DZ UK; 2 Institute of Food, Nutrition and Health, University of Reading, New Agriculture Building, Earley Gate, Whiteknights Road, Reading, RG6 6EU UK; 3 Institute for Cardiovascular and Metabolic Research, School of Biological Sciences, Earley Gate, University of Reading, Reading, RG6 6AS UK; 4 The University of Malawi, P.O Box 280, Zomba, Malawi; 5 School of Food and Nutritional Sciences, University College Cork, T12 N1FK Cork, Ireland

**Keywords:** Pulses, CVD risk markers, Nutrient intake, NDNS, EAT Lancet Index score, Family Food module

## Abstract

**Purpose:**

Diets high in pulses and legumes have been associated with improved cardiovascular disease (CVD) risk markers but the relationship is less well studied in UK populations. To address this, associations between consumption of pulses (dried beans, peas and lentils) and legumes (pulses, fresh peas and green beans) with nutrient intake and status, a sustainable diet quality score (EAT-Lancet index), CVD risk markers and food expenditure was assessed in representative UK populations.

**Methods:**

A secondary analysis of data from the UK National Diet and Nutrition Survey (2008–2019) and the Living Costs and Food Survey (2001–2022) was conducted. To assess the relationships, regression models controlling for covariates were used.

**Results:**

Children and adults consumed mean ± SD 10.6 ± 27.0 g/day and 15.0 ± 21.0 g/day of pulses, and 16.7 ± 32.5 g/day and 27.3 ± 26.0 g/day of legumes, respectively. Diets rich in pulses and legumes were associated with higher intakes of energy, fibre, vitamin E, thiamine, folate, biotin, sodium, potassium, phosphorus, magnesium, iron, zinc, and manganese; lower intakes of saturated fats, total and free sugars and higher plasma selenium and total carotenoid concentrations (all P < 0.05). Consumption of a portion (80 g) of pulses and legumes was associated with a 3.7 point increase in EAT-Lancet index (P < 0.001). Average expenditure on pulses and legumes/person/week in 2022 was £1.68 and £2.90, equivalent to 0.33% and 0.56% of weekly income respectively.

**Conclusions:**

Pulse and legume-rich diets are broadly associated with a more optimum nutrient intake, higher micronutrient status and a more sustainable diet. Strategies are needed to increase pulse and legume consumption in UK populations.

**Supplementary Information:**

The online version contains supplementary material available at 10.1007/s00394-025-03611-2.

## Introduction

A healthful plant-based dietary pattern that encourages a higher consumption of nutrient dense unprocessed plant foods such as fruits, vegetables, legumes, and wholegrains and a lower consumption of animal-based foods, processed foods and free sugars, is associated with improved cardiometabolic health [1]. The WHO recommends consumption of a minimum of 400 g/day of fruits and vegetables [2], supported by epidemiological studies which have reported a 6–10% reduction in risk of cardiovascular diseases (CVD) for every 80 g increase in consumption [3]. The UK government adapted this recommendation to 5 portions of a variety of fruits and vegetables/day with a portion defined as 80 g for fresh and 30 g for dried foods [4]. Despite the launch of this dietary recommendation in 2003, the mean consumption of fruits and vegetables in UK adults was 296 g/day (approximately 3.7 portions) in 2018, considerably below these recommendations, particularly in populations in the most deprived areas [5].

Due to their health benefits, the UK Eatwell guide recommends consumption of 80 g/day of pulses as protein sources, which also contributes 1 portion to the 5 a day recommendation [4]. Pulses are the dried edible seeds of plants from the Leguminosae family (legumes, which are flowering plants with seeds contained within pods), whereas, legumes also include fresh peas, green beans, soya beans and peanuts. Pulses are rich sources of proteins (21–25%), complex carbohydrates (60–65%), dietary fibre (10–20%) and micronutrients such as folate, thiamine, riboflavin, vitamin B6, niacin, iron, zinc, magnesium and potassium [6]. Higher consumption has been associated with lower total and LDL cholesterol [7], and a lower odds of developing hypertension in a UK population [8], with dietary fibre associated with 16% lower all-cause mortality and 18% lower CVD mortality [9]. Furthermore, substituting red meat with pulses was associated with a lower risk of colorectal cancer, type 2 diabetes, and ischaemic heart disease in a Danish population [10].

In 2022, Henry Dimbleby published Part 2 of the National Food strategy which identified the need to move to a more sustainable and healthful diet. Pulses as a food group, are associated with reduced greenhouse gas emissions, water and land use when compared with diets rich in meat [11] and therefore are an important component of an environmentally sustainable diet. In 2019 the EAT Lancet commission, a group of global experts from 16 countries working on defining the targets for healthful and sustainable diets, released a report that recommended consuming 50 g/day (dry weight) of peas, beans and lentils as part of a healthful environmentally friendly diet [12]. A higher EAT-Lancet index has been positively associated with lower risk of developing cardiometabolic diseases [13, 14], improved environmental impact [13] and higher probability of meeting micronutrient recommendations [15]. Although 6.1% (233,000 hectares per year) of the UK’s croppable area is used for growing faba beans and green peas, the majority of beans consumed in the UK are imported, primarily from Brazil, Canada, USA, Kenya and the Netherlands [16]. A report from the BeanMeals project in 2023 found that fresh beans and peas were more commonly purchased compared to legumes in their dried form. Higher purchase of processed alternatives such as baked beans (mean 79 g/day) compared to other canned beans (mean 24 g/day) were also reported possibly due to the convenience [16].

There is limited UK data on pulse or legume-rich dietary patterns, nutrient intakes and status, and CVD risk markers. Since a specific dietary recommendation exists in the UK for pulses [4], this analysis of the National Diet and Nutrition Survey (NDNS) aimed to determine the associations of both pulses (defined here as dried beans, lentils, peas and soy) and legumes (defined here as pulses, green beans and peas) consumption separately in a representative UK population and how this is associated with nutrient intake and status, a sustainable diet quality score (EAT Lancet Index) and health outcomes. To identify the trends in consumer purchasing of pulses and legumes in the UK the family food module dataset from the Living Costs and Food Survey (LCFS) was analysed. We hypothesised that pulse and legume rich diets will be associated with a more beneficial nutrient intake and status, diet quality, health biomarkers and sustainability, than pulse and legume poor diets.

## Methods

### Sources of data for the cross-sectional analysis

The NDNS is a UK government cross-sectional continuous rolling program that began data collection in 2008. The survey collects detailed quantitative data on the diet and nutrient intake, and nutrient status of a representative sample of the UK population aged 1.5 years and above, who reside in private households [17]. A sample of 1000 participants are surveyed each year, with a distribution of 500 children aged 1.5 years to 18 years and 500 adults aged 19 years and above. This paper reports on data collected from the first 11 years of the survey, between 2008 and 2019 which used the same dietary assessment method. The participant sample (n = 15,655) was drawn randomly from postcode address files that were compiled by the national post office and to achieve equality in sampling, households were divided into five primary sampling units (PSUs) with similar sociodemographic characteristics that were sorted by population density [17]. During these years, data collection was conducted in the participants household in two stages; 1) the interviewer stage and 2) the nurse stage, carried out 2 to 4 months apart. During the interviewer stage, participants completed questionnaires which collected information on sociodemographic characteristics, lifestyle, anthropometrics and an estimated three or four-day food diary (7999 adults and 7656 children) [17]. In the nurse stage, blood biomarkers (4181 adults and 2014 children), 24 h urine (3246 adults and 2318 children), blood pressure (4443 adults and 3254 children) [17], waist and hip circumferences (5721 adults and 2302 children), and infant length and mid upper arm circumferences (2278 children) were taken in a subset of the participants. Details of the NDNS methodology has been described in detail elsewhere [17].

### Dietary data

Three or four-day estimated food diaries were used to collect dietary intake data over two or three consecutive weekdays and one weekend day. The diet diaries were manually coded into the Diet in Nutrients Out dietary assessment system that is linked to a food composition database in the NDNS nutrient data bank. For dietary intake data that was reported as raw weights by the participants, a weight change factor based on comparable recipes in McCance and Widdowson’s the Composition of Food series was applied to determine the cooked weight of the food items. Details on the dietary intake methodology are described in NDNS appendices [18]. In this analysis, pulses are defined as all dried beans and their products including soya beans and soy products (such as soya milk and tofu). This was chosen as it matched the NDNS variable categorisation defined as “Beansg” in the database. We also analysed a further category called legumes, which included the original pulse data, as well as fresh peas, green beans, and fresh beans such as faba beans which are classed as vegetables (commonly called broad beans) when eaten fresh. These categories include intake data from composite dishes. All results are presented as mean consumption in g/day for all participants that recorded at least three days of dietary data.

### Anthropometric measures, cardiovascular disease risk markers and biochemical analytes of nutrient status

The data used in our analysis included anthropometrics (body mass index, waist circumference, waist to hip ratio), CVD risk markers (diastolic blood pressure, systolic blood pressure, plasma glucose, total cholesterol, HDL-cholesterol, LDL-cholesterol, triacylglycerol and C-reactive protein), and nutrient status markers (plasma ferritin, plasma iron, red blood cell thiamine, red blood cell riboflavin, plasma vitamin B6, serum vitamin B12, serum holotranscobalamin, serum folate, plasma vitamin C, plasma retinol, plasma total carotenoids, plasma alpha tocopherol, 25-hydroxy-vitamin D, plasma selenium and zinc in years 1–10 and serum selenium and zinc in year 11 and urinary sodium). Portable stadiometers were used for height measurement for all participants apart from older adults aged 65 + years with whom demispan was used. These methods are reported elsewhere [17, 18].

### Sustainable diet quality assessment using the EAT-Lancet index

The EAT-Lancet index used in this analysis was developed and validated by Stubbendorff et al. [19]. The adapted dietary index has a total possible score of 42 points derived from 14 food components; participants have a score ranging from 0 to 3 points indicating low and high adherence for each component respectively. The 14 food components include wholegrains, vegetables, fruits, potatoes, nuts, legumes, fish, poultry, pork, beef and lamb, eggs, dairy, unsaturated fats and added sugar [19]. The EAT-Lancet index recommendations are based on dry weights of certain foods. The NDNS reports food as consumed hence, we converted food eaten to raw weights by using conversion factors for wholegrains, legumes, meat, and fish using McCance and Widdowson food composition tables [20]. In the wholegrains food component, baked goods made from wholegrain flours were divided by 0.18, for grains such as rice by 0.39 and for wholegrain breakfast cereals by 0.92. For red meat (pork, lamb, and beef), the total grams consumed/day was divided by 0.72, for poultry by 0.75, for white fish by 0.83, for oily fish by 0.88 and for shellfish by 0.67. For all pulses, the total grams consumed/day was divided by 2.83. The foods included in the 14 categories are listed in Supplementary Table 1. Added fats were calculated by summing unsaturated fats (monounsaturated fats, omega 3 fatty acids and omega 6 fatty acids), as reported by the NDNS in the person level database, and the total amount includes unsaturated fats consumed from composite dishes. Free sugar was calculated by subtracting monosaccharides from fruits and vegetables from total monosaccharides consumed as reported in the NDNS food level database.

### Consumer expenditure using the living costs and food survey

The LCFS is an annual continuous cross-sectional survey of household expenditure on goods and services and household income in the UK. The survey samples approximately 5400 participants aged 7 and above annually, who are requested to keep a record of all their daily expenditures made over two weeks. The sample is representative of the UK population as the participants are selected from the postal address file which have been divided into approximately 700 PSUs which are weighted to factor in demographic distributions [21]. For this analysis, we used the family food module dataset of the LCFS which includes a record of food and non-alcoholic drink expenditures made by the household, including food purchased for consumption outside the home from 2001–2002 to 2021–2022 [22]. The family food module reports household expenditure in gross income quintiles and equalised income deciles. Expenditure was reported in pence per person per week (p/person/week) for each food item ranging from meat to dairy and bread [22].

### Statistical analysis

All statistical analyses were performed using Stata software, version 18.0 (Stata Corp LP). The NDNS database was analysed as a survey using the svy command in Stata. Combined survey weights for all 11 years, statistical subpopulation 4 (defined as strata 4) and PSUs were applied to the database prior to analysis to ensure a true representation of the UK population, reduce biases due to non-response and differences in socioeconomic status and total population per cluster as recommended by the NDNS user guide [23]. Individual weights were applied in the analysis of nutrient intake and EAT-Lancet index score; while nurse weights were applied in the analysis of the association of pulse and legume consumption with blood pressure and anthropometric measurements, and blood weights were applied in the analysis of the association of pulse and legume consumption with all blood analyte variables such as lipids and glucose [23]. Linearised survey adjusted linear regression was used to assess the association of pulse consumption with dietary intake, CVD risk markers, nutrient status markers and EAT Lancet index score. The models were controlled for age, sex, energy, supplement intake, household income, ethnicity, region of residence and year of survey with the exception of the demographic characteristics which were controlled for total dietary energy. Pulse consumption was analysed as a continuous variable in all models. The analysis was run separately for children and adults. A P-value of < 0.05 was considered statistically significant. All data is presented as mean and standard deviation (SD). To analyse the trends in expenditure of pulses and legumes in LCFS, descriptive statistics using excel were conducted and reported in percentages and means in £/person/week.

## Results

### Comparison of pulses and legumes consumption across demographic characteristics

**Fig. 1:**
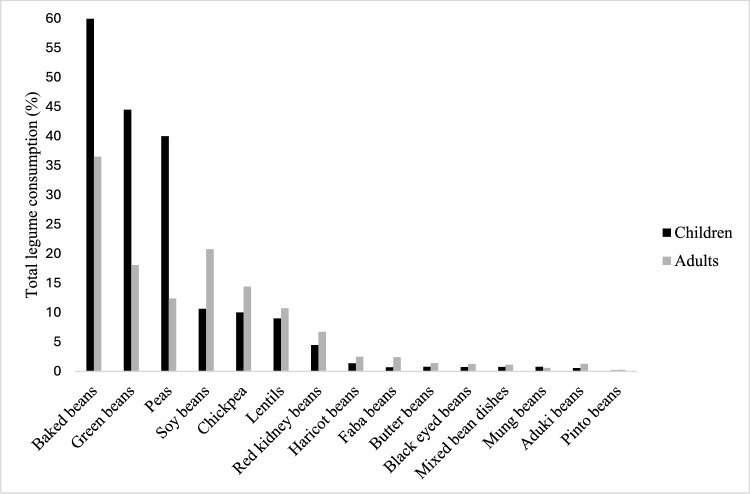
Percentage distribution of the commonly consumed legumes in children (1.5–18 years) and adults (19–96 years) in the UK from the NDNS (2008–2019). Haricot beans refers to all haricot beans consumed in other forms other than baked beans

Pulses were consumed by 55.1% (n = 4221/7656) and 56.7% (n = 4537/7999) of the UK children (1.5–18 years) and adults (19–96 years), which represented mean intakes of 10.6 ± 27.0 g/d and 15.0 ± 21.0 g/d respectively. Only 1% (n = 60/7656) of the children and 2% (n = 140/7999) of the adults included in the analysis consumed on average one portion (80 g) or more of pulses/day. The percentage of participants who consumed legumes (defined here as beans, peas, and green beans) was 72.5% (n = 5548/7656) in children and 79% (n = 6319/7999) in adults and the mean consumption was 16.7 ± 32.5 g/day and 27.3 ± 26.0 g/day in children and adults respectively. Only 1.4% (n = 110/7656) of children and 5.2% (n = 416/7999) of adults consumed on average one portion of legumes/day. In children, baked beans were the most consumed pulse followed by green beans, peas, soybeans and chickpeas. In adults, baked beans were also the most consumed pulse, followed by soybeans, green beans, chickpeas and peas (Fig. 1**)**.

The mean pulse and legume consumption by different demographic characteristics is reported in Table 1. Those of Asian ethnicity, 4.6% of the total sample population (n = 721/15655), consumed significantly more pulses (8.1 g-10.4 g/day in children and 7.5 g-27.5 g/day in adults) and legumes (8.5 g-11.6 g/day in children and 10.8 g-25.9 g/day in adults) than all other ethnicities, P < 0.001. Individuals from Northern Ireland consumed significantly less pulses (1.6–4.3 g/day in children and 2.0–5.5 g/day in adults) and legumes (0.9–7.4 g/day in children and 4.0–8.6 g/day in adults) compared to individuals from the rest of the UK (all p < 0.05). In adults, participants from year 9 (2016–2017) consumed 5.3 g more legumes compared to participants from year 3 (2010–2011) (P = 0.046). Children from households with an annual income under £5,000 consumed less pulses than children from households with an annual income that ranged from £5,000-£9,999, (P = 0.038) but not for legumes (P = 0.49). Compared to adults from households with an annual income between £25,000-£29,999, only adults from households from £15,000-£19,999 and £45,000–49,999 consumed more pulses (P < 0.05) but these differences were not statistically significant for legumes. In years 2 (2009–2010) and 3 (2010–2011) of the survey, children consumed 3.0 g (P = 0.029) and 2.5 g (p = 0.039) more pulses compared to year 7 (2014/2015), respectively; while in adults, participants from year 9 (2016–2017) consumed 4.6 g (P = 0.041) and 3.8 g (P = 0.027) more pulses compared to years 2 (2009–2010) and 6 (2013–2014) respectively, and participants from year 11 (2018–2019) consumed 4.6 g (P = 0.047) and 3.8 g (P = 0.026) more pulses compared to years 2 (2009–2010) and 6 (2013–2014) respectively. In children, participants from years 2 (2009–2010) and 3 (2010–2011) consumed 3.0 g to 4.8 g more legumes compared to participants from years 4 (2011–2012), 6 (2013–2014), 7 (2014–2015), and 8 (2015–2016) (P < 0.05).

**Table 1:** Intake of pulses and legumes according to demographic characteristics among UK children (n = 7656) and adults (n = 7999) from the NDNS (2008–2019)

Characteristics	Children (n = 7656)						Adults (n = 7999)					
	Pulses			Legumes			Pulses			Legumes		
	Mean ± SD g/day	Coefficient per portion	P value	Mean ± SD g/day	Coefficient per portion	P value	Mean ± SD g/day	Coefficient per portion	P value	Mean ± SD g/day	Coefficient per portion	P value
Age^a,c^	10.6 ± 27.2	0.02	0.807	16.5 ± 30.1	0.01	0.903	15.0 ± 21.1	−1.0	< 0.001	26.9 ± 23.1	0.06	0.018
**Sex**												
Males^a,b^	11.2 ± 28.0	–	–	17.3 ± 32.9			16.6 ± 22.4	–	–	29.4 ± 26.6	–	–
Females	10.0 ± 26.3	− 0.3	0.585	16.0 ± 32.0	−0.1	0.862	13.4 ± 18.5	−0.3	0.698	25.3 ± 24.4	0.5	0.605
Ethnicity												
White	9.5 ± 24.5	−11.8	< 0.001	16.0 ± 31.1	−9.28	< 0.001	13.2 ± 17.4	−26.2	< 0.001	26.1 ± 23.8	−21.8	< 0.001
Mixed ethnicity	8.9 ± 19.9	− 12.0	< 0.001	14.1 ± 23.4	−10.6	< 0.000	15.6 ± 15.4	−23.4	< 0.001	22.1 ± 17.1	−25.4	< 0.001
Black or Black British	11.1 ± 26.0	− 9.9	< 0.001	14.2 ± 28.3	−10.7	< 0.001	14.4 ± 17.2	−24.2	< 0.001	20.1 ± 19.1	−26.9	< 0.001
Asian or Asian British^a,b^	20.8 ± 35.7	–	–	24.5 ± 38.5	–	–	39.1 ± 37.6	–	–	47.6 ± 38.1	–	–
Any other group	12.3 ± 32.1	−8.2	0.011	16.4 ± 33.9	−7.8	0.022	28.3 ± 26.4	10.2	0.110	40.1 ± 30.6	−7.5	0.275
Region												
North England	10.9 ± 22.8	4.0	< 0.001	15.8 ± 26.5	4.7	< 0.001	12.9 ± 14.8	2.3	0.008	25.8 ± 20.6	5.5	< 0.001
Central England/Midlands	11.1 ± 23.0	4.2	< 0.001	18.6 ± 28.0	7.2	< 0.001	15.1 ± 19.2	3.9	0.001	28.8 ± 22.7	8.1	< 0.001
South England including London	10.8 ± 23.5	3.9	< 0.001	17.2 ± 27.7	5.9	< 0.001	16.4 ± 19.4	5.3	< 0.001	28.6 ± 23.2	8.1	< 0.001
Scotland	8.6 ± 31.0	1.6	0.072	12.2 ± 43.4	1.7	0.185	13.6 ± 23.1	3.1	0.014	24.2 ± 30.0	4.3	0.007
Wales	11.2 ± 45.2	4.3	< 0.001	18.7 ± 51.6	7.3	< 0.001	15.2 ± 31.0	4.6	< 0.001	29.4 ± 38.2	9.6	< 0.001
Northern Ireland^a,b^	6.9 ± 41.7	–	–	11.3 ± 51.1	–	–	10.9 ± 30.1	–	–	20.2 ± 41.2	–	–
Household income £/annum												
Under 5,000^a^	9.4 ± 20.6	–	–	14.4 ± 30.1	–	–	15.3 ± 20.7	4.3	0.151	24.4 ± 26.4	2.4	0.518
5,000—9,999	12.6 ± 34.8	3.8	0.053	17.8 ± 40.8	4.3	0.131	13.2 ± 19.8	2.7	0.183	23.7 ± 25.6	2.4	0.378
10,000—14,999	10.4 ± 27.8	1.5	0.395	15.4 ± 34.5	1.9	0.490	13.8 ± 17.7	3.0	0.119	26.9 ± 23.9	5.2	0.056
15,000—19,999	10.2 ± 31.3	1.1	0.503	16.4 ± 36.4	2.4	0.328	15.1 ± 22.7	4.1	0.028	29.4 ± 27.9	7.4	0.005
20,000—24,999	10.3 ± 29.8	1.4	0.466	16.5 ± 32.6	2.8	0.288	14.7 ± 19.5	3.3	0.236	27.5 ± 26.5	4.9	0.113
25,000—29,999^b^	10.5 ± 28.9	1.6	0.383	18.2 ± 35.7	4.6	0.097	11.6 ± 15.4	–	–	22.9 ± 21.1	–	–
30,000—34,999	10.4 ± 28.5	1.1	0.520	16.9 ± 39.1	2.6	0.324	13.4 ± 15.1	2.1	0.238	26.3 ± 22.1	3.8	0.133
35,000—39,999	9.3 ± 22.7	0.2	0.914	16.0 ± 27.3	2.1	0.415	13.3 ± 15.6	1.9	0.366	26.3 ± 21.4	3.7	0.227
40,000—44,999	11.3 ± 26.5	1.9	0.328	17.0 ± 34.7	2.6	0.363	17.6 ± 17.4	6.1	0.060	27.2 ± 20.7	4.2	0.242
45,000—49,999	9.2 ± 24.2	−0.2	0.906	16.0 ± 30.7	1.6	0.531	14.5 ± 16.1	3.1	0.044	26.9 ± 19.6	4.2	0.126
50,000—74,999	9.7 ± 26.2	0.2	0.881	16.1 ± 31.4	1.6	0.501	14.8 ± 13.2	3.0	0.144	27.7 ± 20.9	4.7	0.058
75,000—99,999	11.3 ± 25.8	1.9	0.315	18.5 ± 31.6	4.0	0.146	13.7 ± 13.2	2.1	0.083	23.9 ± 18.6	0.9	0.784
100,000 or more	11.5 ± 26.0	2.1	0.293	19.6 ± 33.5	5.1	0.080	17.8 ± 18.2	5.9	0.092	29.3 ± 22.0	6.0	0.164
Year of survey												
2008–2009	10.0 ± 25.4	−1.2	0.279	17.7 ± 33.7	−0.6	0.988	14.0 ± 21.1	−8.0	0.013	27.3 ± 27.9	−7.5	0.030
2009–2010	10.9 ± 30.9	0.01	0.991	18.7 ± 37.8	−0.8	0.372	12.5 ± 16.5	−9.4	0.003	25.0 ± 22.8	−9.6	0.003
2010–2011	11.5 ± 29.9	0.6	0.651	18.8 ± 35.5	−0.6	0.347	13.3 ± 20.1	−8.1	0.010	24.8 ± 24.2	−9.3	0.004
2011–2012	10.9 ± 31.5	0.04	0.977	15.8 ± 35.2	−0.5	0.425	13.2 ± 21.2	−8.5	0.007	25.8 ± 28.1	−8.6	0.007
2012–2013	10.4 ± 23.4	−0.5	0.657	17.0 ± 31.6	−0.9	0.880	15.5 ± 17.0	−6.2	0.049	28.8 ± 22.0	−5.6	0.101
2013–2014	9.6 ± 22.4	−0.5	0.353	14.5 ± 26.7	−0.7	0.116	13.7 ± 16.0	−7.8	0.012	25.7 ± 21.8	−8.4	0.009
2014–2015	9.1 ± 20.0	−1.6	0.159	14.6 ± 24.8	−0.8	0.129	14.8 ± 21.8	−6.9	0.040	26.8 ± 26.7	−7.7	0.031
2015–2016	10.2 ± 29.8	−0.3	0.848	15.4 ± 33.2	−0.7	0.476	15.4 ± 17.6	−6.1	0.054	26.9 ± 24.3	−7.2	0.027
2016–2017^b^	10.2 ± 23.8	−0.3	0.835	16.1 ± 27.3	−0.3	0.779	21.6 ± 33.4	–	–	34.3 ± 35.3	–	–
2017–2018	12.9 ± 33.7	2.5	0.222	18.0 ± 37.0	−0.6	0.504	13.5 ± 17.6	−7.8	0.013	26.9 ± 23.5	−7.0	0.035
2018–2019^a^	10.5 ± 22.4	–	–	16.6 ± 28.7	–	–	16.8 ± 18.0	−4.7	0.140	28.0 ± 22.1	6.2	0.059

The coefficient values reported are from regression models with energy, sex, age, income, region, ethnicity, supplement use and survey year as covariates as appropriate for the demographic characteristic

^a^ indicates the regression analysis base factor variable that was used to compare with the other factor variables within that grouping in children

^b^ indicates the regression analysis base factor variable that was used to compare with the other factor variables within that grouping in adults

^c^ Age was analysed as a continuous variable

### Association of pulse and legume intake with dietary intake and health outcomes

#### Nutrient intake and status

In children, consumption of pulses and legumes was associated with significantly higher intakes of dietary energy, fibre, vitamin E, thiamine, folate, biotin, sodium, potassium, phosphorus, magnesium, iron, zinc, and manganese; and lower intakes of saturated fats, total and free sugars (all P < 0.05), (Table 2**)**. Only a higher intake of protein was observed for greater legume consumption (P < 0.001). However, consuming pulses was not significantly associated with circulating biomarkers of nutrient intake, with the exception of plasma vitamin B6 which was 14.4 nmol/L lower per portion (80 g) of pulses consumed (P = 0.012). One portion of legumes was associated with a higher serum selenium and plasma total carotenoids, (P < 0.05) see Table 3.

**Table 2:** Association between consumption of one portion (80 g/day) of pulses and legumes with nutrient intakes among children (n = 7656) and adults (n = 7999) in the NDNS from years 2008–2019

Nutrients	Children					Adults				
	Mean ± SD	Pulses		Legumes		Mean ± SD	Pulses		Legumes	
		coefficient per portion	P value	coefficient per portion	P value		coefficient per portion	P value	coefficient per portion	P value
Energy (kcal/day)	1575 ± 812	224	< 0.001	235	< 0.001	1819 ± 430	137	< 0.001	162	< 0.001
Energy (kJ/day)	6517 ± 2775	608	0.001	848	< 0.001	7701 ± 1801	584	< 0.001	504	< 0.001
Protein (g/day)	58.1 ± 33.5	2.4	0.037	4.8	< 0.001	73.8 ± 18.9	4.0	0.001	1.6	0.198
Total fat (g/day)	59.2 ± 35.7	−0.8	0.264	−2.4	0.004	67.4 ± 19.7	−4.0	< 0.001	−4.0	< 0.001
Saturated fat (g/day)	22.9 ± 14.7	−1.6	0.006	−1.6	0.001	25.2 ± 8.3	−2.4	< 0.001	−3.2	< 0.001
Carbohydrate (g/day)	214 ± 113	0.08	0.973	0.05	0.973	221 ± 55	11.2	< 0.001	16.8	< 0.001
Free Sugars (g/day)	64.3 ± 61.3	−9.6	< 0.001	−9.6	< 0.001	58.5 ± 29.7	−8.0	< 0.001	−6.2	0.001
AOAC Fibre (g/day)	14.6 ± 8.6	7.2	< 0.001	7.2	< 0.001	18.3 ± 4.9	7.2	< 0.001	9.6	< 0.001
**Vitamins**										
Vitamin E (mg/day)	7.70 ± 4.96	0.8	0.003	0.24	0.201	9.41 ± 3.32	0.8	0.036	0.7	0.008
Thiamine (mg/day)	1.27 ± 0.71	0.08	0.002	0.2	< 0.001	1.48 ± 0.45	0.2	< 0.001	0.2	< 0.001
Riboflavin (mg/day)	1.40 ± 0.92	0.0032	0.943	0.004	0.923	1.59 ± 0.55	0.04	0.279	0.0064	0.885
Niacin (mg/day)	27.0 ± 15.9	−1.6	0.037	0.56	0.402	35.3 ± 10.8	0.4	0.538	1.0	0.251
Vitamin B12 (mg/day)	4.04 ± 4.66	−0.16	0.317	−0.05	0.735	5.32 ± 3.00	−0.2	0.260	−0.6	0.006
Folate (μg/day)	184 ± 114	42.4	0.026	45.6	< 0.001	246 ± 83	44.8	< 0.001	44.8	< 0.001
Pantothenic acid (mg/day)	5.01 ± 3.28	0.03	0.837	0.2	0.290	5.76 ± 1.94	0.24	0.030	0.2	0.102
Biotin (mg/day)	23.8 ± 18.0	3.2	< 0.001	2.4	0.004	34.6 ± 14.8	2.0	0.034	3.8	0.002
Vitamin C (mg/day)	77.5 ± 73.2	−6.4	0.114	3.2	0.326	82.0 ± 45.3	11.2	< 0.001	8	0.041
**Minerals**										
Sodium (mg/day)	1763 ± 1046	322	< 0.001	174	< 0.001	2109 ± 656	172	< 0.001	288	< 0.001
Potassium (mg/day)	2127 ± 1004	330	< 0.001	368	< 0.001	2811 ± 708	386	< 0.001	384	< 0.001
Calcium (mg/day)	766 ± 461	32.8	0.140	1.6	0.948	815 ± 258	27.2	0.058	56.2	0.003
Magnesium (mg/day)	192 ± 94	35.2	< 0.001	37.6	< 0.001	260 ± 74	40.0	< 0.001	47.2	< 0.001
Phosphorus(mg/day)	996 ± 483	72.8	< 0.001	80.8	< 0.001	1228 ± 314	81.6	< 0.001	80	< 0.001
Total iron (mg/day)	8.43 ± 4.69	1.6	< 0.001	1.6	< 0.001	10.4 ± 3.0	2.16	< 0.001	2.6	< 0.001
Zinc (mg/day)	6.49 ± 3.61	0.5	0.012	0.002	< 0.001	8.48 ± 2.39	0.6	< 0.001	0.8	< 0.001
Manganese (mg/day)	2.16 ± 1.87	0.5	0.002	0.64	< 0.001	3.11 ± 1.10	0.7	< 0.001	1.0	< 0.001
**Food groups**										
Vegetables (g/day)	100 ± 93	118	< 0.001	120	< 0.001	189 ± 85	138	< 0.001	120	< 0.001
Red meat (g/day)	46.4 ± 54.3	−1.5	0.670	2.4	0.494	63.9 ± 39.0	11.2	0.003	5.6	0.047
Total meat (g/day)	75.2 ± 69.6	12	0.007	−4.8	0.252	101 ± 50	22.4	< 0.001	12.8	< 0.001
Dairy^a^ (g/day)	116 ± 217	7.9	0.452	64	0.444	76.0 ± 76.9	4	0.627	2.4	0.663
EAT-Lancet index score										
Index score	21.5 ± 5.6	3.7	< 0.001	3.8	< 0.001	22.2 ± 3.6	3.7	< 0.001	3.7	< 0.001

The coefficient values reported are from regression models with energy, sex, age, income, region, ethnicity, supplement use and survey year as covariates

^a^Dairy was defined as whole milk or derivative equivalents, regular milk, 1% fat milk, semi skimmed milk, skimmed milk, yoghurt and other fermented milk products, hard cheese, soft cheese, cream, butter, butter-based spreads

**Table 3:** Association between the consumption of one portion (80 g/day) of pulses and legumes with plasma markers nutrient intake among children |(n = 7656) and adults (n = 7999) in the NDNS from years 2008–2019

Outcome	Children					Adults				
	Mean ± SD	Pulses		Legumes		Mean ± SD	Pulses		Legumes	
		coefficient per portion	P value	coefficient per portion	P value		coefficient per portion	P value	coefficient per portion	P value
Plasma Ferritin (μg/L)	38.1 ± 32.0	−2.40	0.636	−1.36	0.699	105 ± 78	−1.60	0.861	2.4	0.650
Plasma Iron (μg/L)	−0.24 ± 0.47	−0.03	0.434	−0.04	0.299	−0.14 ± 0.26	−0.02	0.409	−0.001	0.967
Serum Vitamin B12 (ng/L)	338 ± 143	−21.60	0.175	−6.88	0.594	275 ± 78	−4.80	0.625	−5.76	0.438
Serum Holo-transcobalamin (pmol/L)	74.9 ± 30.4	−3.40	0.650	−3.50	0.596	68.6 ± 16.9	−0.008	0.947	2.60	0.432
Serum Vitamin D (nmol/L)	46.4 ± 24.3	−2.40	0.488	−0.05	0.983	46.1 ± 15.8	−0.16	0.940	−0.40	0.799
Plasma Vitamin C (μmol/L)	59.4 ± 23.6	−6.40	0.050	−0.32	0.920	51.1 ± 15.6	2.40	0.218	2.08	0.135
Plasma Selenium (μmol/L)	0.93 ± 0.16	0.08	0.668	0.0048	0.011	1.06 ± 0.14	0.32	0.986	0.008	0.370
Plasma zinc (μmol/L)	14.1 ± 2.33	−0.40	0.376	−0.40	0.272	13.5 ± 1.66	−0.24	0.640	−0.072	0.790
Serum Folate (nmol/L)	18.3 ± 12.1	−1.60	0.392	−0.24	0.864	19.8 ± 15.9	0.48	0.665	0.72	0.460
Plasma total Carotenoids (μmol/L)	2.08 ± 0.84	0.16	0.326	0.27	0.021	2.08 ± 0.64	0.24	0.044	0.16	0.016
Plasma Retinol (μmol/L)	1.36 ± 0.41	0.16	0.998	0.02	0.732	1.88 ± 0.34	−0.02	0.732	−0.016	0.612
Plasma alpha Tocopherol (μmol/L)	21.7 ± 9.1	−0.96	0.319	−0.72	0.473	27.6 ± 5.12	−0.16	0.826	−0.15	0.757
Red blood cell thiamine	1.11 ± 0.06	0.01	0.188	0.002	0.673	1.11 ± 0.04	0.00	0.639	0.005	0.226
Red blood cell riboflavin (ng/L)	1.42 ± 0.23	−0.01	0.893	−0.016	0.463	1.35 ± 0.13	0.02	0.271	0.006	0.644
Plasma Vitamin B6 (nmol/L)	63.5 ± 40.5	−14.40	0.012	−8.0	0.183	56.9 ± 31.9	5.36	0.200	1.52	0.635
Urinary sodium (mmol/L)	102 ± 85	21.6	0.204	10.4	0.317	137 ± 37	4.0	0.645	0.16	0.983

The coefficient values reported are from regression models with energy, sex, age, income, region, ethnicity, supplement use and survey year as covariates

In adults, consumption of pulses and legumes was related to significantly higher intakes of dietary energy, fibre, carbohydrates, vitamins E and C, thiamine, folate, biotin, sodium, potassium, phosphorus, magnesium, iron, zinc and manganese; and lower intakes of saturated fats, free sugars, vitamin B12 and haem iron (all P < 0.05). A higher intake of protein was only observed for legumes (P = 0.001), see Table 2**.** A portion (80 g) of pulses and legumes were significantly associated with higher plasma carotenoids, but serum markers of nutrient status were not significantly associated, see Table 3.

### CVD risk markers

Pulse and legume consumption were not significantly related to anthropometric or CVD risk markers in either children or adults as shown in Table 4.

**Table 4:** Association between consumption of one portion (80 g/day) of pulses and legumes with CVD risk markers among children (n = 7656) and adults (n = 7999) in the NDNS from years 2008–2019

Outcome	Children					Adults				
		Pulses		Legumes			Pulses		Legumes	
	Mean ± SD	Coefficient per portion	P value	Coefficient per portion	P value	Mean ± SD	Coefficient per portion	P value	Coefficient per portion	P value
BMI (kg/m^2^)	21.7 ± 6.8	−0.07	0.796	−0.07	0.796	27.3 ± 3.6	0.08	0.709	0.08	0.709
Waist circumference (cm)	75.4 ± 17.8	0.08	0.956	0.08	0.956	92.4 ± 9.9	0.80	0.410	0.80	0.410
Waist to hip ratio	0.80 ± 0.11	0.005	0.414	0.005	0.414	0.88 ± 0.06	0.001	0.884	0.001	0.884
Systolic blood pressure (mmHg)	112 ± 17.7	−0.24	0.833	−0.24	0.833	126 ± 11.6	0.40	0.701	0.40	0.701
Diastolic blood pressure (mmHg)	62.8 ± 13.7	−0.28	0.767	−0.28	0.767	73.2 ± 7.4	0.32	0.600	0.32	0.600
Plasma glucose (mmol/L)	4.84 ± 0.54	−0.02	0.178	−0.02	0.178	5.37 ± 0.87	0.01	0.879	0.01	0.879
Total cholesterol (mmol/L)	4.05 ± 0.83	0.02	0.818	0.02	0.818	5.00 ± 0.75	−0.08	0.275	−0.08	0.275
LDL^a^ Cholesterol (mmol/L)	2.29 ± 0.72	0.06	0.451	0.06	0.451	3.01 ± 0.65	−0.06	0.351	−0.06	0.351
HDL^b^ Cholesterol (mmol/L)	1.41 ± 0.37	−0.01	0.823	−0.01	0.823	1.44 ± 0.30	−0.03	0.172	−0.03	0.172
Triacylglycerol (mmol/L)	0.80 ± 0.46	−0.05	0.178	−0.05	0.178	1.23 ± 0.45	−0.02	0.752	−0.02	0.752
C-reactive protein (mg/L)	2.06 ± 3.20	0.72	0.251	0.72	0.251	3.74 ± 4.18	−0.32	0.382	−0.32	0.382

The coefficient values reported are from regression models with energy, sex, age, income, region, ethnicity, supplement use and survey year as covariates

^a^LDL (Low density lipoprotein)

^b^HDL (Low density lipoprotein)

### EAT-Lancet Index

In children, the mean score for the Eat-Lancet index was 21.5 ± 5.2 ranging from 9.0 to 36.0. Consumption of 1 g of pulses and legumes was associated with a 0.05 increase in the EAT Lancet index score (P < 0.001); this translates into a 3.7 and 3.8 point higher score for pulses and legumes per portion (80 g) respectively, see Table 2**.**

In adults, the mean score for the Eat-Lancet index was 22.3 ± 3.4 ranging from 8.0 to 38.0. Consuming 1 g of pulses and legumes was associated with a 0.05 increase in the EAT-Lancet index score (P < 0.001); this translates into a 3.7 point higher score per portion (80 g) for both pulses and legumes, see Table 2**.**

### Household expenditure on pulses and legumes in the UK

**Fig. 2:**
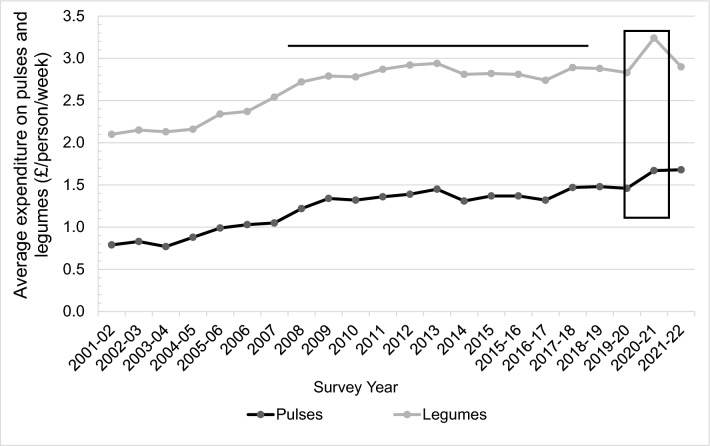
The absolute spend in pounds (£)/person/week (**A**) and percentage of weekly income spent (**B**) on pulses and legumes across 10 income deciles between 2008 to 2018–19

**Fig. 3:**
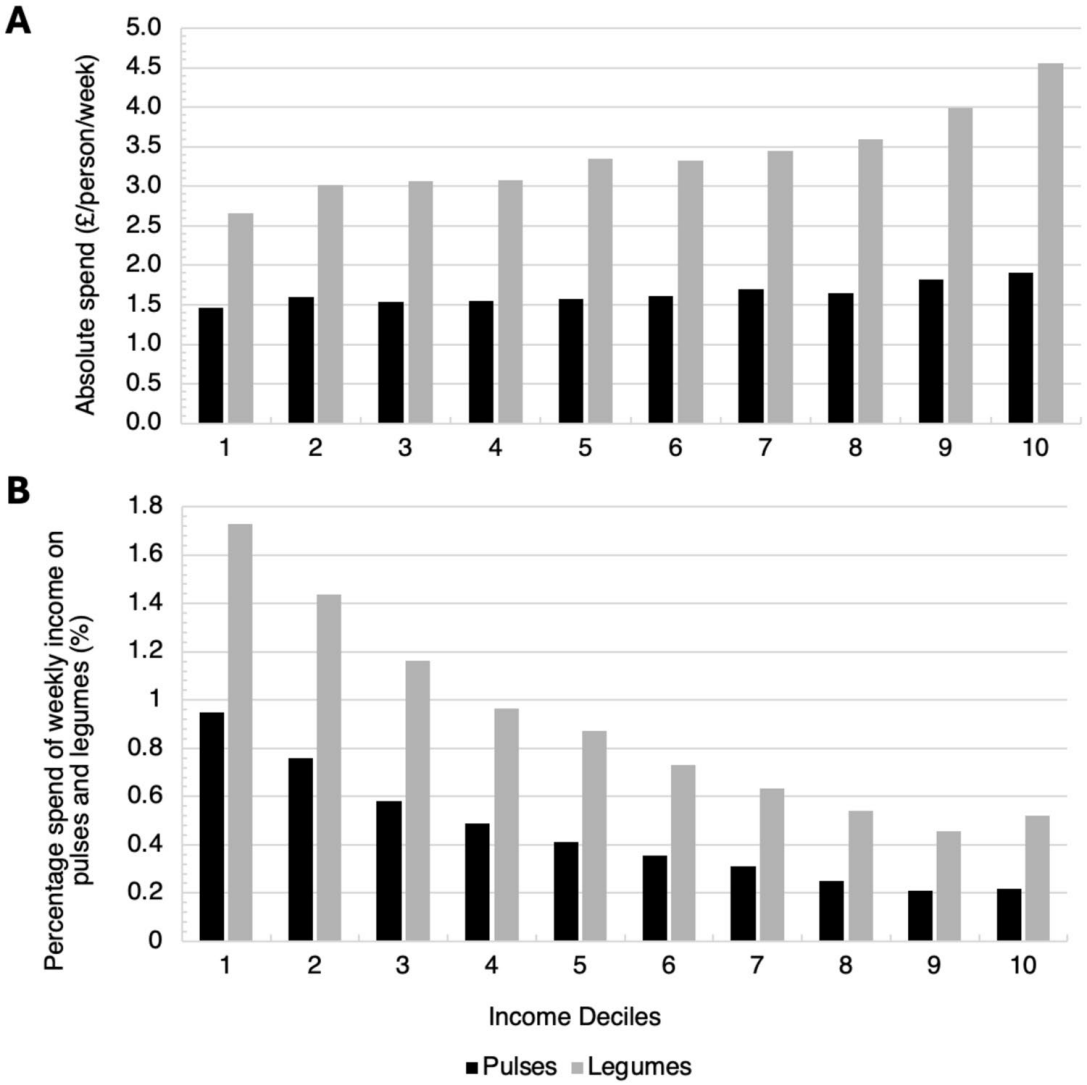
The absolute spend in pounds (£)/person/week (A) and percentage of weekly income spent (B) on pulses and legumes across 10 income deciles between 2008 to 2018–19

A total of 163,692 people were surveyed in the family food module of the LCFS since 2001 with each decile of income/week comprising of between 14,400 to 17,300 participants. There was a general increasing trend in the average household expenditure/person in pulses and legumes from £0.79 to £1.68 and £2.10 to £2.90 respectively between 2001 and 2022 (Fig. 2). An average of 0.49% to 0.62% of the total household expenditure was spent on pulses and legumes between 2001 to 2021–22 across deciles 1 to 10 of income in the UK. Households from deciles 1 had the lowest absolute spend on pulses and legumes compared to all the other deciles while decile 10 had the highest absolute spend (Fig. 3a). However, as a percentage of weekly income, deciles 1 and 10 spent 0.95% and 0.22% on pulses and 1.73% and 0.52% on legumes, respectively (Fig. 3b).

Legumes are depicted by the gray line and pulses by the black line. The horizonal line is the time span of the NDNS; The black box represents the COVID-19 pandemic. The differences in the changes in reporting of the year were due to changes in the family food module from financial year basis to calendar year basis and back to financial year basis.

The average equalised disposable pre-income tax (OECD Scale) per week for each decile (averaged from 2010–2020/21) was1 £154; 2 £210; 3 £264; 4 £319; 5 £383; 6 £454; 7 £544; 8 £664, 9 £875; 10 £875 +.

## Discussion

These data analyses have shown that in the UK, diets rich in pulses and legumes were associated with a more optimal nutrient intake and a higher EAT-Lancet index in both children and adults, but not CVD risk markers relative to pulse and legume poor diets. Ethnicity, region, year of the survey and household income were sociodemographic factors found to impact on this relationship, with average household expenditure on pulses and legumes shown to increase by £1/person/week between 2001 and 2022.

As expected diets higher in pulses and legumes were associated with higher micronutrient and dietary fibre intakes and lower total fats, saturated fats and free sugar. Furthermore, a higher Eat-Lancet Index was observed with greater pulse and legume intake, which was reflective of a more sustainable and higher quality diet. Micronutrient deficiencies persist in the UK with iron, folate, and zinc being the most common deficiencies especially in the most vulnerable populations [24–27]. Similar to our findings, diets of individuals that consumed ≥ 66.3 g/day of pulses were associated with higher intakes of thiamine, folate, niacin, iron, zinc, calcium, magnesium, phosphorus, selenium and potassium, compared to non-consumers in American [28], Australian [29], and Canadian populations [30]. While higher micronutrient intakes were observed in our analysis, only plasma total carotenoids were associated with pulse and legume intake in adults, and vitamin C and selenium were associated with legume intake in children. These observations could reflect the higher consumption of vegetables in those who consumed higher pulses and legumes, as plasma carotenoids can be used as a biomarker of vegetable consumption [31]. Adults who consumed higher amounts of pulses also had a lower consumption of all meat, and since meat is a rich source of vitamin B12 this could be a contributing factor for the observed lower vitamin B12 intake. However, these lower intakes were not reflected in plasma vitamin B12, a biomarker of intake and vitamin B12 status. The lower dietary vitamin B12 intakes in the NDNS population supports the findings from a Canadian population where estimates were lower in adults that consumed > 66.3 g/day of pulses versus non-consumers [28]. Higher fibre intakes have also been linked with diets higher in pulses and legumes not only in the UK (6.0–22.9 g/day) but also in the Australian (8.5 g/day) [29], Canadian (5.6 g-14.1 g/day) [28] and American populations (4.3 g-21.8 g/day) (19), compared to non-consumers, which is unsurprising as pulses and legumes have a high fibre content [6].

In contrast to the beneficial relationship between diets higher in pulses and legumes and dietary fibre and micronutrients, our analysis also revealed a positive relationship with total dietary energy and sodium intakes, a finding supported by previous studies in western populations [30] [28]. Pulses and legumes are often consumed as part of composite dishes that are carbohydrate based (e.g. rice, tacos and tortillas) and energy dense [28, 30, 32], which could be contributing to the positive association between dietary patterns that contain higher pulses and energy intake. Although total energy was higher, there were no significant associations between pulse or legume-rich diets and anthropometric measures. This apparent inconsistency may relate to different lifestyle characteristic, such as a higher physical activity level in those consuming pulse or legume-rich diets and warrants further investigation. Our finding of a positive association between dietary sodium intake and pulse consumption is not unique to the UK population. In a Canadian cohort high salt intake was proposed to be related to a higher consumption of canned beans, Hispanic bean dishes and soups that traditionally have a high sodium content [28]. Our analysis revealed that baked beans (canned haricot beans in a tomato sauce), which have a relatively high sodium content (around 340 mg/80 g), were the most commonly consumed pulse in the UK in both children and adults. However, estimation of sodium intake from diet diary analysis is challenging and generally underestimates consumption. A key limitation is that salt added during cooking or at the table, an important source of dietary sodium, is not included in the estimation. Within the NDNS, urinary sodium is used as an objective biomarker of sodium intake. In contrast to the dietary sodium data, there was no association between pulse and legume-rich diets and urinary sodium, suggesting no significant relationship between pulses, or legumes and sodium intake. Furthermore, despite high sodium intakes being associated with increased risk of developing hypertension [33], we did not find an associations between consumption of pulses, or legumes and blood pressure in adults or children.

Research suggests that the prevalence of cardiovascular events can be lowered by reducing the amount of saturated fats in the diet and replacing these fats with unsaturated fats [34]. Although pulse and legume-rich diets were associated with lower saturated fat and free sugar consumption in both children and adults, we did not observe a relationship with CVD risk markers. Previous studies have reported associations between pulse consumption and improved CVD risk, with lower total cholesterol, LDL cholesterol, TAG and body weight and a higher HDL cholesterol [35]. Ramdath et al. [36] reported that diets of individuals that consumed at least 150 g of pulses per week were associated with improved postprandial blood glucose response and lower fasting blood cholesterol and consuming 1000 g or more pulses per week was related to further improvements in glycated haemoglobin, fasting blood glucose and insulin concentrations, which suggests a dose-dependent relationship with cardiometabolic disease risk markers.

An overall increasing trend in expenditure for both pulses and legumes was found in UK households from 2001–02 to 2021–22 using data from Family Food module, which followed a similar pattern to their mean consumption from 2008 to 2019 in the NDNS. The reasons for the higher pulse and legume consumption in participants from year 9 (2016–2017) and 11 (2018–2019) when compared to some earlier years are not clear. However, it is of note, that there were specific global campaigns promoting pulse consumption over this period, including 2016 declared as the International Year of Pulses, and February 10th as World Pulse Day from 2018 by the United Nations which may have impacted on dietary intakes [37]. In the Family Food Module the linear trend in pulse expenditure seen in this analysis could also be due to the rising cost of beans, particularly brand names that are popular in the UK [23]. Those in the highest income decile were shown to spend the smallest percentage of their income on pulses and legumes probably reflecting the higher weekly income. Pulses offer a cheaper source of dietary protein than meat and cost is an important consideration in food choice especially in disadvantaged communities facing food insecurity and the rise in cost of living [38]. The sharp increase in expenditure on pulses and legumes observed in 2020–21 could be due to the COVID-19 pandemic lockdowns in the UK (first lockdown on 23rd March 2020) as people bought more food to cook at home and stockpiling dry foods, including tinned foods, became more common.

The strengths of these analysis were that the NDNS and LCSF are representative population of children and adults living within the UK. Furthermore, disaggregated composite dishes into individual ingredients, including meats, fruits and vegetables, provided a more detailed picture of an individual’s dietary intake and allowed correct grouping of the data into 14 food groups. Limitations of this analysis are that NDNS is a cross-sectional study, hence it cannot prove causation, but only associations. It should also be noted that our analysis did not include sub-group analysis according to dietary practices such as vegetarianism and veganism which could have impacted on the strength of the relationship with the study outcome measures. Furthermore, the EAT-Lancet index is based on raw weights of certain foods while the NDNS mainly reported foods as consumed, which required conversion factors to estimate raw weights from cooked weights. It was also unclear whether the pulses bought were consumed and contributed to dietary intake from the LCSF. Furthermore, the LCFS (household level) did not provide the disaggregated values for fruits and vegetables.

In conclusion, our analysis indicates that diets rich in pulses and legumes in children and adults residing in the UK were associated with higher intakes of key nutrients and a higher EAT-Lancet index. These findings support the recommendation to include pulses as part of a healthful and sustainable diet. However, the consumption of legumes, and particularly pulses, is low in all population groups within the UK and strategies to increase their consumption are required. In addition to increasing pulses and legumes within individual dishes, inclusion of pulse flours within staple foods, such as bread or pasta, is a feasible option [39]. This approach has been reported to reduce glycaemic response and improve satiety compared with conventional foods [40] [41] and reduces the need for significant dietary change. Evaluation of the efficacy of these strategies to improve human and environmental health, and beneficial dietary behaviour change is urgently needed.

## Supplementary Information

Below is the link to the electronic supplementary material.

Supplementary file1 (DOCX 19 KB)

## Data Availability

Data described in the manuscript can be freely accessed from the UK Data services website [39]. The datasets used and analysed are available from the corresponding author on reasonable request.

## Abbreviations

BBSRC: Biotechnology and Biological Sciences Research Council
BMI: Body Mass Index
CVD: Cardiovascular Diseases
HDL: High Density Lipoprotein
LDL: Low Density Lipoprotein
LCFS: Living Costs and Food Survey
NDNS: National Diet and Nutrition Survey
NHS: National Health Service
PSU: Primary Sampling Units
REC: Research Ethics Committee
SD: Standard Deviation
TAG: Triacylglycerol
SACN: Scientific Advisory Committee for Nutrition
USA: United States of America
UK: United Kingdom
WHO: World Health Organisation

## References

[1] Satija A, Hu FB (2018) Plant-based diets and cardiovascular health. Trends Cardiovasc Med 28:437–441. 10.1016/j.tcm.2018.02.004 29496410 10.1016/j.tcm.2018.02.004 PMC6089671

[2] World Health Organisation Increasing fruit and vegetable consumption to reduce the risk of noncommunicable diseases. https://www.who.int/tools/elena/interventions/fruit-vegetables-ncds. Accessed 31 Oct 2023

[3] Lock K, Pomerleau J, Causer L et al (2005) The global burden of disease attributable to low consumption of fruit and vegetables: implications for the global strategy on diet. Bull World Health Organ 83:100–108 15744402 PMC2623811

[4] United Kingdom Government Healthy eating: applying All Our Health. In: GOV.UK. https://www.gov.uk/government/publications/healthy-eating-applying-all-our-health/healthy-eating-applying-all-our-health. Accessed 10 Aug 2023

[5] Health Survey for England Fruit & vegetables. In: Health Survey for England. http://healthsurvey.hscic.gov.uk/data-visualisation/data-visualisation/explore-the-trends/fruit-vegetables.aspx. Accessed 20 May 2024

[6] Singh N (2017) Pulses: an overview. J Food Sci Technol 54:853–857. 10.1007/s13197-017-2537-4 28303036 10.1007/s13197-017-2537-4 PMC5336460

[7] Padhi EMT, Ramdath DD (2017) A review of the relationship between pulse consumption and reduction of cardiovascular disease risk factors. J Funct Foods 38:635–643. 10.1016/j.jff.2017.03.043

[8] Hartley M, Fyfe CL, Wareham NJ et al (2022) Association between Legume Consumption and Risk of Hypertension in the European Prospective Investigation into Cancer and Nutrition (EPIC)-Norfolk Cohort. Nutrients 14:3363. 10.3390/nu14163363 36014869 10.3390/nu14163363 PMC9415844

[9] Veronese N, Solmi M, Caruso MG et al (2018) Dietary fiber and health outcomes: an umbrella review of systematic reviews and meta-analyses. Am J Clin Nutr 107:436–444. 10.1093/ajcn/nqx082 29566200 10.1093/ajcn/nqx082

[10] Fabricius FA, Thomsen ST, Fagt S, Nauta M (2021) The health impact of substituting unprocessed red meat by pulses in the Danish diet. Eur J Nutr 60:3107–3118. 10.1007/s00394-021-02495-2 33515322 10.1007/s00394-021-02495-2

[11] Dimbleby H (2022) The National Food Strategy - The Plan. In: National Food Strategy. https://assets.publishing.service.gov.uk/media/61684fe3e90e071979dfec4a/national-food-strategy-the-plan.pdf. Accessed 2 Jun 2024

[12] Willett W, Rockström J, Loken B et al (2019) Food in the Anthropocene: the EAT-Lancet Commission on healthy diets from sustainable food systems. Lancet 393:447–492. 10.1016/S0140-6736(18)31788-4 30660336 10.1016/S0140-6736(18)31788-4

[13] Knuppel A, Papier K, Key TJ, Travis RC (2019) EAT-Lancet score and major health outcomes: the EPIC-Oxford study. The Lancet 394:213–214. 10.1016/S0140-6736(19)31236-X 10.1016/S0140-6736(19)31236-X 31235280

[14] Cacau LT, Benseñor IM, Goulart AC et al (2023) Adherence to the EAT-Lancet sustainable reference diet and cardiometabolic risk profile: cross-sectional results from the ELSA-Brasil cohort study. Eur J Nutr 62:807–817. 10.1007/s00394-022-03032-5 36266476 10.1007/s00394-022-03032-5

[15] Hanley-Cook GT, Argaw AA, de Kok BP et al (2021) EAT–Lancet diet score requires minimum intake values to predict higher micronutrient adequacy of diets in rural women of reproductive age from five low- and middle-income countries. BJN 126(1):92–100 10.1017/S0007114520003864 32993824

[16] Nicholson W, Jones K (2023) Putting Beans on the plate: Analysis of UK demand and supply of beans and plant based proteins

[17] Venables MC, Roberts C, Nicholson S et al (2022) Data Resource Profile: United Kingdom National Diet and Nutrition Survey Rolling Programme (2008–19). Int J Epidemiol 51:e143–e155. 10.1093/ije/dyac106 35640141 10.1093/ije/dyac106 PMC9365634

[18] Public Health England (2021) Appendix A Dietary data collection and editing for Year 10 and 11 of the NDNS RP. In: UK Data Archive Study. https://api.repository.cam.ac.uk/server/api/core/bitstreams/b555ca0e-9c14-4571-b68c-9d7084209895/content. Accessed 24 Jul 2023

[19] Stubbendorff A, Sonestedt E, Ramne S et al (2022) Development of an EAT-Lancet index and its relation to mortality in a Swedish population. Am J Clin Nutr 115:705–716. 10.1093/ajcn/nqab369 34791011 10.1093/ajcn/nqab369 PMC8895215

[20] McCance RA, Widdowson EM (2014) McCance and Widdowson’s The Composition of Foods. Royal Society of Chemistry

[21] Office of National Statistics (2023) Living Costs and Food Survey QMI - Office for National Statistics. https://www.ons.gov.uk/peoplepopulationandcommunity/personalandhouseholdfinances/incomeandwealth/methodologies/livingcostsandfoodsurveyqmi#methods-used-to-produce-the-living-costs-and-food-survey-lcf-data. Accessed 1 Nov 2023

[22] Defra statistics (2023) Family Food 2020/21. In: GOV.UK. https://www.gov.uk/government/statistics/family-food-202021/family-food-202021. Accessed 1 Nov 2023

[23] Public Health England (2021) National Diet and Nutrition Survey Years 9–11 (2016/17–2018/19) User Guide. In: UK Data Archive. https://assets.publishing.service.gov.uk/government/uploads/system/uploads/attachment_data/file/943114/NDNS_UK_Y9-11_report.pdf. Accessed 24 Jul 2023

[24] Public Health England (2021) National Diet and Nutrition Survey Rolling programme Years 9 to 11 (2016/2017 to 2018/2019). 29

[25] Pollock RF, Muduma G (2017) A budget impact analysis of parenteral iron treatments for iron deficiency anemia in the UK: reduced resource utilization with iron isomaltoside 1000. ClinicoEconom Outcomes Res 9:475–483. 10.2147/CEOR.S139525 10.2147/CEOR.S139525 PMC5557122 28848355

[26] Thane CW, Bates CJ, Prentice A (2004) Zinc and vitamin A intake and status in a national sample of British young people aged 4–18 y. Eur J Clin Nutr 58:363–375. 10.1038/sj.ejcn.1601792 14749759 10.1038/sj.ejcn.1601792

[27] Derbyshire E (2018) Micronutrient Intakes of British Adults Across Mid-Life: A Secondary Analysis of the UK National Diet and Nutrition Survey. Front Nutr 5:55 30073167 10.3389/fnut.2018.00055 PMC6060686

[28] Mudryj AN, Yu N, Hartman TJ et al (2012) Pulse consumption in Canadian adults influences nutrient intakes. Br J Nutr 108:S27–S36. 10.1017/S0007114512000724 22916812 10.1017/S0007114512000724

[29] Grains & Legumes Nutrition Council (2011) Legumes and Nutrition. In: Grains & Legumes Nutrition Council. https://www.glnc.org.au/resource/legumes-nutrition/. Accessed 31 Jul 2023

[30] Mitchell DC, Lawrence FR, Hartman TJ, Curran JM (2009) Consumption of Dry Beans, Peas, and Lentils Could Improve Diet Quality in the US Population. J Am Diet Assoc 109:909–913. 10.1016/j.jada.2009.02.029 19394480 10.1016/j.jada.2009.02.029

[31] Campbell DR, Gross MD, Martini MC et al (1994) Plasma carotenoids as biomarkers of vegetable and fruit intake. Cancer Epidemiol Biomark Prev 3:493–500 8000300

[32] Lane L, Reynolds C, Wells R (2023) Beans, Peas and Pulses: UK consumption patterns and the impact of recipes

[33] Grillo A, Salvi L, Coruzzi P et al (2019) Sodium Intake and Hypertension. Nutrients 11:1970. 10.3390/nu11091970 31438636 10.3390/nu11091970 PMC6770596

[34] Hooper L, Martin N, Jimoh OF et al (2020) Reduction in saturated fat intake for cardiovascular disease. Cochrane Database Syst Rev. 10.1002/14651858.CD011737.pub3 32428300 10.1002/14651858.CD011737.pub2 PMC7388853

[35] Anderson JW, Major AW (2002) Pulses and lipaemia, short- and long-term effect: Potential in the prevention of cardiovascular disease. Br J Nutr 88:263–271. 10.1079/BJN2002716 10.1079/BJN2002716 12498626

[36] Ramdath D, Renwick S, Duncan AM (2016) The Role of Pulses in the Dietary Management of Diabetes. Can J Diabetes 40:355–363. 10.1016/j.jcjd.2016.05.015 27497151 10.1016/j.jcjd.2016.05.015

[37] Calles T, Del Castello R, Baratelli M et al (2019) The International Year of Pulses. Environ Earth Sci 78:1–8

[38] Johnstone A, Lonnie M, undefined, (2023) The cost-of-living crisis is feeding the paradox of obesity and food insecurities in the UK. Obesity (Silver Spring) 31:1461–1462. 10.1002/oby.23740 37203335 10.1002/oby.23740 PMC10947515

[39] Lovegrove JA, O’Sullivan DM, Tosi P et al (2023) ‘Raising the Pulse’: The environmental, nutritional and health benefits of pulse-enhanced foods. Nutr Bull 48:134–143. 10.1111/nbu.12601 36649740 10.1111/nbu.12601 PMC10947378

[40] Bajka BH, Pinto AM, Perez-Moral N et al (2023) Enhanced secretion of satiety-promoting gut hormones in healthy humans after consumption of white bread enriched with cellular chickpea flour: A randomized crossover study. Am J Clin Nutr 117:477–489. 10.1016/j.ajcnut.2022.12.008 36811474 10.1016/j.ajcnut.2022.12.008 PMC10131617

[41] Goñi I, Valentín-Gamazo C, (2003) Chickpea flour ingredient slows glycemic response to pasta in healthy volunteers. Food Chem 81:511–515. 10.1016/S0308-8146(02)00480-6

