# Supplementary material for: Pulse and legume consumption is associated with a more optimal nutrient intake and a higher EAT-Lancet index in a representative UK population
Source: Eur J Nutr. 2025 Mar 26;64(3):139. doi: 10.1007/s00394-025-03611-2 (PMC11937138; doi:10.1007/s00394-025-03611-2)
Supplement: Supplementary file 1 — Supplementary file1 (DOCX 19 KB) [file 394_2025_3611_MOESM1_ESM.docx]

Pulse and legume consumption is associated with a more optimal nutrient intake and a higher EAT-Lancet index in a representative UK population.

Yankho Kaimila^1,2,4^ (0000-0002-1650-9050), Oyinkansola A. Olotu^1,2^(0009-0003-9949-4249), Miriam E. Clegg^1,5^ (0000-0002-8871-0116), Kim G. Jackson^1,2,3^ (0000-0002-0070-3203) and Julie A. Lovegrove^1,2,3^ (0000-0001-7633-9455)

^1^ Hugh Sinclair Unit of Human Nutrition, Harry Nursten Building, Pepper Lane, University of Reading Reading, RG6 6DZ. UK

^2^ Institute of Food, Nutrition and Health, Harry Nursten Building, Pepper Lane, University of Reading Reading, RG6 6DZ. UK

^3^Institute for Cardiovascular and Metabolic Research, Harry Nursten Building, Pepper Lane, University of Reading, Reading, RG6 6DZ. UK

^4^The University of Malawi, P.O Box 280, Zomba. Malawi

^5^Present address: School of Food and Nutritional Sciences, University College Cork, Ireland

Correspondence: Julie Lovegrove: [j.a.lovegrove@reading.ac.uk](mailto:j.a.lovegrove@reading.ac.uk)

**Supplemental Table 1** Description of food components from the NDNS (2008-2019) included in the EAT-Lancet index score calculation

| **Components in the EAT-Lancet diet index.** | |
| --- | --- |
| Whole grains | Brown granary bread, whole meal bread, oatmeal bread, rye bread, soya and linseed bread, Gluten free brown bread, oatmeal bread, bran loaf, whole meal teacakes, rye crispbread, multigrain crispbread, digestive biscuits with wheatgerm, digestive biscuits with oats, nestle oats and more cereal bars, nutrigrain elevenses bars, oat flapjacks dipped in chocolate, oatcakes, rolled oats, oatmeal, oatmeal cookies and biscuits, oat bran, Weetabix breakfast biscuits, Weetabix oaty bars, whole meal biscuits, whole meal crackers, quinoa, barley, bran wheat, brown Basmati rice, brown rice, red rice, brown rice noodles, buckwheat, bulgur wheat, cornmeal flour, cornmeal porridge, corn flour, whole meal flour, brown flour, whole meal flour, rye flour, couscous, millet, wheatgerm, fibre-rich breakfast cereals (≥10% fibre), whole meal spaghetti,  We divided flour products by 0.18 to get whole grain contribution in the food. Cooked wholegrains were divided by 0.39 and oatmeal by 0.92 to get raw weights. Conversion factors are based on previous work. |
| Potatoes | Boiled potatoes, fried potatoes, deep fried potatoes, potatoes included in dishes such as potato salad, potato scones and other meals made from potato flour. Cassava and cassava meals such as gari. |
| Vegetables | All vegetables except legumes. |
| Fruits | Fruits and berries, including dried fruits |
| Dairy | Whole milk or derivative equivalents. Regular milk, 1% fat milk, semi skimmed milk, skimmed milk, yoghurt and other fermented milk products, hard cheese, soft cheese, cream, butter, butter-based spreads. |
| Beef and lamb | Beef, lamb, minced meat with pork and lamb, processed meats with beef and lamb including sausages. |
| Pork | Pork, minced meat of pork, processed meats with pork including ham, bacon, and sausages. |
| Chicken | Chicken, turkey, duck, goose, and other poultry. |
| Eggs | Boiled eggs, fried eggs and eggs in dishes such as omelette and pie. |
| Fish | Oily fish, white fish, fish products, shellfish. To obtain raw weights, oily fish, white fish and shellfish were divided by 0.88, 0.83 and 0.67 respectively. Conversion factors are based on previous work |
| Legumes | Dry beans, lentils, peas, soy, and soy derived products. Targets and index refer to raw weight which was calculated from cooked weights by dividing the cooked weight by 2.83 based off previous work. |
| Nuts | Peanuts or tree nuts. All nuts and seeds including peanuts, nut mixes such as almond paste. |
| Unsaturated oils | All plant oils and plant margarines. |
| Added sugar | Sucrose and monosaccharides except sugars in fruits and vegetables |
